# Prevalence, clinical management and risks associated with acute faecal incontinence in the critical care setting: the FIRST questionnaire survey

**DOI:** 10.1186/cc9900

**Published:** 2011-03-11

**Authors:** R Binks

**Affiliations:** 1 Airedale NHS Foundation Trust, Bradford, UK

## Introduction

The FIRST survey was designed to examine the prevalence, awareness and management of acute faecal incontinence with diarrhoea (AFI), and the clinical challenges associated with AFI for healthcare professionals (HCP) in the critical care setting.

## Methods

A descriptive cross-sectional survey. Data were collected from ICUs or critical care units in Germany, Italy, Spain and the UK using a questionnaire. The questionnaire contained 20 questions for completion by HCP, and six specific questions for hospital pharmacists or purchasing personnel. Questions concerned the epidemiology, awareness and management of AFI, and associated clinical issues. Analysis of the results was conducted so that respondents remained anonymous.

## Results

A total of 960 questionnaires were completed (Germany *n *= 200; Italy *n *= 261; Spain *n *= 267; UK *n *= 232) by nurses (60%), physicians (29%) and pharmacists or purchasing personnel (11%). Estimated prevalence of AFI ranged from 9 to 37% of patients on the day of the survey. The majority of respondents reported a moderately low awareness of the clinical challenges associated with AFI and its prioritisation in their units. Patients with AFI commonly had compromised skin integrity (perineal dermatitis, moisture lesions or sacral pressure ulcers). Reducing the risk of cross-infection and protecting skin integrity were rated as the most important clinical challenges. Forty-nine per cent responded that they had no hospital protocol or guideline for the management of AFI. There was generally low awareness of nursing time spent managing AFI episodes by some hospital personnel, but 60% of respondents estimated that 10 to 20 minutes are required for managing an AFI episode, requiring two or three healthcare staff. The key reported benefits of faecal management systems included: reduced risk of cross-contamination and infection, reduced risk of skin breakdown, and improved patient comfort and dignity. In those not using a faecal management system, the main reason reported was lack of availability or that devices were not included in the hospital guidelines.

## Conclusions

AFI in the critical care setting may be an underestimated problem that is associated with a high use of nursing time. In many institutions there is a lack of protocols or guidelines, which might improve the management of AFI in the critical care setting.

